# Real-Time Pyruvate Chemical Conversion Monitoring Enabled by PHIP

**DOI:** 10.1021/jacs.2c13198

**Published:** 2023-03-01

**Authors:** Gabriele Stevanato, Yonghong Ding, Salvatore Mamone, Anil P. Jagtap, Sergey Korchak, Stefan Glöggler

**Affiliations:** † NMR Signal Enhancement Group, Max Planck Institute for Multidisciplinary Sciences, Am Fassberg 11, 37077 Göttingen, Germany; ‡ Center for Biostructural Imaging of Neurodegeneration of the University Medical Center Göttingen, Von-Siebold-Street 3A, 37075 Göttingen, Germany

## Abstract

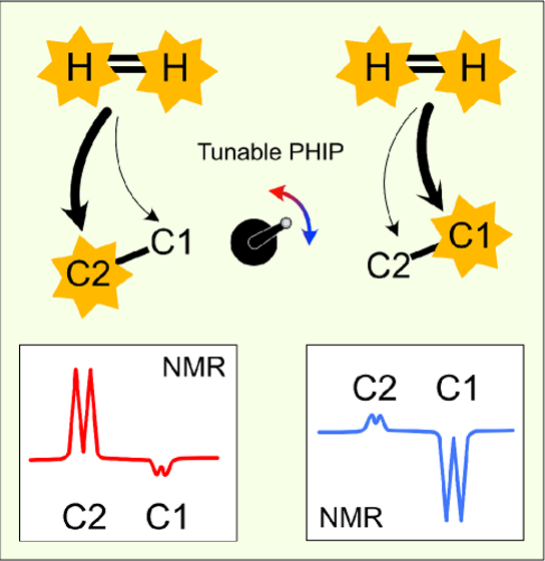

In recent years, parahydrogen-induced polarization side arm hydrogenation (PHIP-SAH) has been applied to hyperpolarize [1-^13^C]pyruvate and map its metabolic conversion to [1-^13^C]lactate in cancer cells. Developing on our recent MINERVA pulse sequence protocol, in which we have achieved 27% [1-^13^C]pyruvate carbon polarization, we demonstrate the hyperpolarization of [1,2-^13^C]pyruvate (∼7% polarization on each ^13^C spin) *via* PHIP-SAH. By altering a single parameter in the pulse sequence, MINERVA enables the signal enhancement of C1 and/or C2 in [1,2-^13^C]pyruvate with the opposite phase, which allows for the simultaneous monitoring of different chemical reactions with enhanced spectral contrast or for the same reaction *via* different carbon sites. We first demonstrate the ability to monitor the same enzymatic pyruvate to lactate conversion at 7T in an aqueous solution, *in vitro*, and in-cell (HeLa cells) *via* different carbon sites. In a second set of experiments, we use the C1 and C2 carbon positions as spectral probes for simultaneous chemical reactions: the production of acetate, carbon dioxide, bicarbonate, and carbonate by reacting [1,2-^13^C]pyruvate with H_2_O_2_ at a high temperature (55 °C). Importantly, we detect and characterize the intermediate 2-hydroperoxy-2-hydroxypropanoate in real time and at high temperature.

## Introduction

Nuclear magnetic resonance (NMR) is a non-invasive and quantitative analytical technique with applications in structural biology,^1^ drug discovery,^2^ and biomedicine.^3^ The limited NMR sensitivity has been tackled by hyperpolarization techniques to enhance signals over 10,000-fold with a large focus on metabolic studies. Dynamic nuclear polarization (DNP), parahydrogen-induced polarization (PHIP^4−7^ and SABRE-signal amplification by reversible exchange^8−12^), and other methods have been intensively researched with this respect.^13−22^ SABRE succeeded in hyperpolarizing [1-^13^C]acetate^23^ and [1-^13^C]pyruvate at >10% in methanol and very recently in water–methanol solution.^24−27^ PHIP is relatively inexpensive and achieves carbon polarization levels similar to those of dissolution DNP on specific targets.^28^ Inter alia, the PHIP side-arm hydrogenation (PHIP-SAH) method by Reineri *et al.*(29) can in principle be applied to any molecule containing a carboxylic group. In essence, a precursor molecule formed by an unsaturated moiety linked through an ester bond to the substrate of interest is hyperpolarized by PHIP, and upon hydrolysis *via* base injection (NaOH or Na_2_CO_3_), the hyperpolarized metabolite is retained.^29,30^ The heteronuclear polarization transfer from parahydrogen is realized by magnetic field cycling (MFC) or pulsed NMR methods.^31−40^

Among the various molecular systems, pyruvate plays a crucial role in deregulated glycolytic pathways in diseases associated with inflammation, neurodegeneration, and cancer.^41,42^ As the end product of glycolysis, pyruvate might be converted into alanine *via* alanine transaminase (ALT) or lactate *via* lactate dehydrogenase (LDH), or it can enter the tricarboxylic acid cycle (TCA) *via* the catalysis of the pyruvate dehydrogenase complex (PDH). The metabolic conversion of [1-^13^C]pyruvate-to-[1-^13^C]lactate (P–L) has shown potential in clinical trials of prostate cancer patients,^43−45^ and we have recently used it to produce the first mouse tumor imaging by PHIP-SAH.^46^ However, by approaching TCA, [1-^13^C]pyruvate is converted *via* PDH into ^13^CO_2_, thereby preventing the direct detection of downstream TCA metabolites. Instead, [2-^13^C]pyruvate enters TCA and can potentially be used to access, for example, [5-^13^C]glutamate, thus broadening the range of accessible metabolic information.^47^

[1,2-^13^C]Pyruvate combines the advantages of [1-^13^C] and [2-^13^C]pyruvates. It has been explored in the context of *in vitro* and *in vivo* DNP^48,49^ and in that of nuclear long-lived spin states.^50,51^ SABRE succeeded in [1,2-^13^C]pyruvate ^13^C hyperpolarization at <2% albeit in methanol-*d*_4_.^52^ In addition to its central role in cellular energy production, pyruvate plays a crucial part in shielding neurons and other cell types from the harmful effects of hydrogen peroxide (H_2_O_2_). The primary cause of the neuroprotective effect appeared to be related to the non-enzymatic decarboxylation of α-ketoacids rather than to an improvement of energy metabolism.^53^

**Figure 1:**
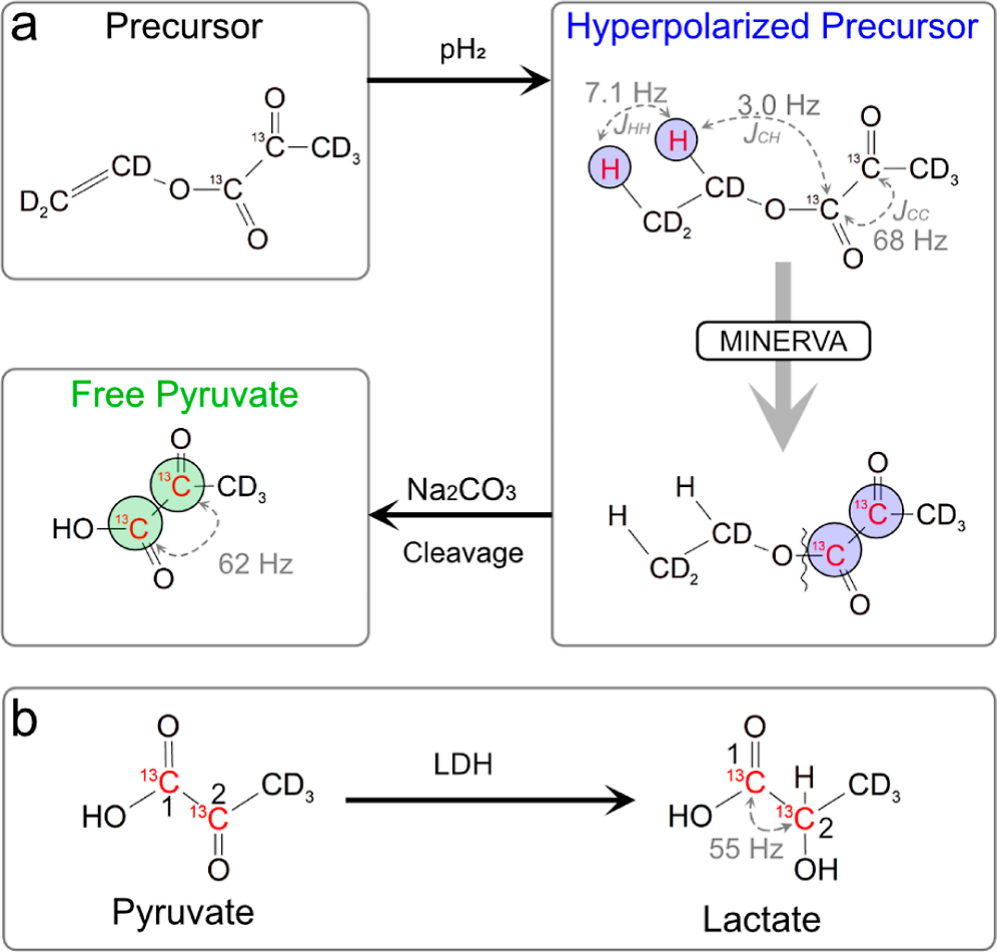
(a) PHIP-SAH steps: [1,2-^13^C]perdeuterated vinyl pyruvate precursor upon parahydrogen addition converts into hyperpolarized ethyl [1,2-^13^C]pyruvate precursor. The MINERVA sequence transfers the polarization to the ^13^C_1_ and/or ^13^C_2_ of the pyruvate moiety. Upon Na_2_CO_3_-induced hydrolysis, free [1,2-^13^C]pyruvate is obtained. (b) Hyperpolarized (HP) P–L conversion triggered by the lactate dehydrogenase (LDH) enzyme. Circles indicate HP nuclei.

**Figure 2:**
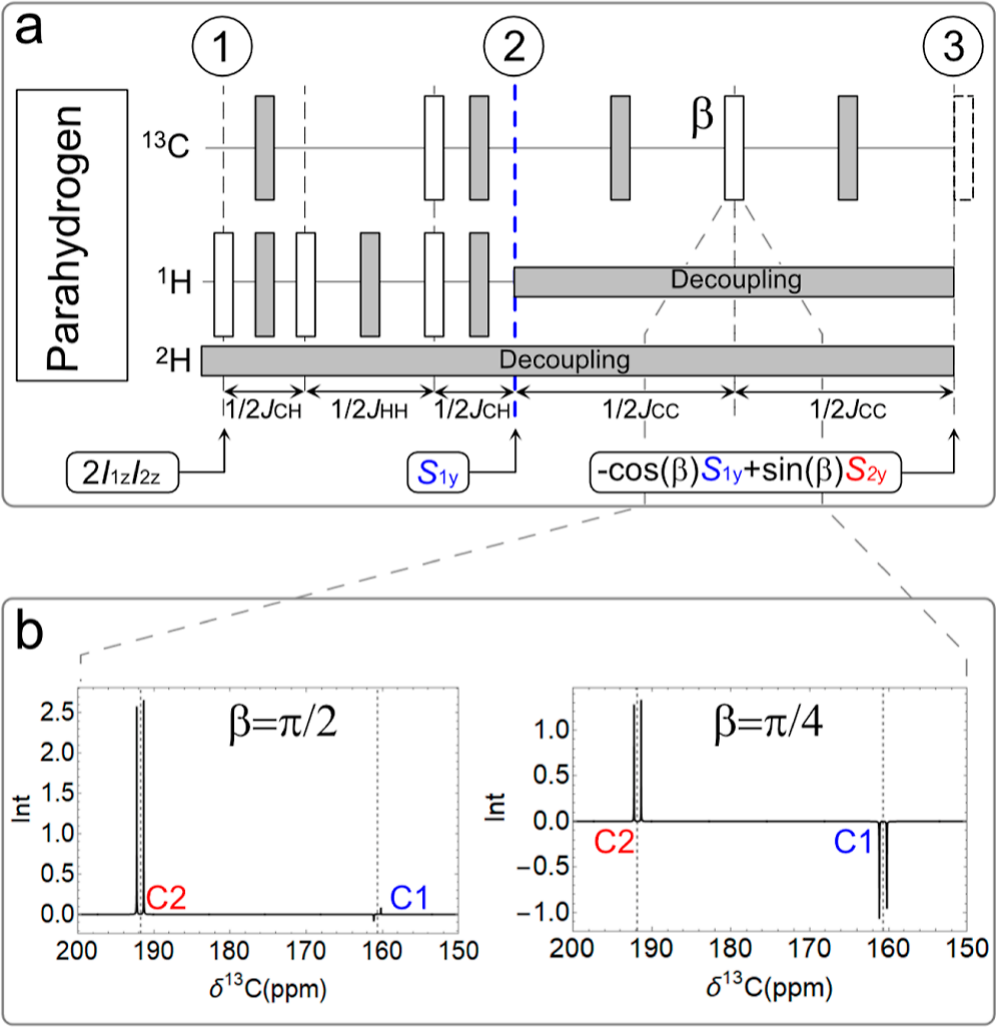
(a) MINERVA for the transfer of longitudinal spin order of parahydrogen into magnetization. The filled and empty rectangles are 180 and 90° pulses, respectively. The phases of the 180° pulses lay along the transverse axes of the rotating plane except for the last optional flip pulse (dashed empty rectangle) on the *x*-axis. The spin density operator at steps 1, 2, and 3 is reported in boxes. The last 90° dashed pulse is removed for direct observation. (b) Simulated spectra with β = π/2 (left) and β = π/4 (right).

Here, we report on [1,2-^13^C]pyruvate hyperpolarization *via* PHIP-SAH in combination with our maximizing insensitive nuclei enhancement reached *via* para-hydrogen amplification (MINERVA) method at 7T.^54^ We control the phase and polarization levels at C1 and C2 carbon positions by only adjusting one sequence parameter: the final β pulse (see Figure 2). Hyperpolarized [1,2-^13^C]pyruvate in an aqueous solution is used to monitor the real-time P–L conversion (Figure 1b) *in vitro* and *in*-*cell*. Furthermore, we show that we can track multiple chemical reactions simultaneously through the different carbon-13-tagged sites by investigating the H_2_O_2_-induced pyruvate decarboxylation pathway. Importantly, we report the transient real-time formation of the intermediate 2-hydroperoxy-2-hydroxypropanoate (**I**) at high temperature (55 °C) and at different pH conditions.

## Results and Discussion

### PHIP-SAH

For all our studies, we used [1,2-^13^C]perdeuterated vinyl pyruvate which is synthesized as described in the Supporting Information(54) and used as a [1,2-^13^C]pyruvate PHIP-SAH precursor (see Figure 1a). Experimentally, parahydrogen at 7 bars is supplied to a degassed 100 μL acetone-*d*_6_ solution containing 5 mM precursor and 10 mM of [1,4-Bis(diphenylphosphino)butane](1,5-cyclooctadiene)rhodium(I) tetrafluoroborate catalyst. The duration of parahydrogen supply was 20 s at 55 °C and 7T. The subsequent application of MINERVA transfers the parahydrogen polarization to the target carbon nuclei on the precursor molecule at C1, C2, or both positions depending on the β angle used (see below). In the following 5 s, the pressure is released, and 100 μL of a 50 mM solution of Na_2_CO_3_ in D_2_O is injected into the NMR tube *via* a plastic cannula (i.d. 1 mm) coupled externally to a 1 mL syringe. Upon injection of the aqueous solution, the drop in catalyst’s solubility initiates the catalyst’s precipitation. Following the base injection, a vacuum pump connected to the NMR tube is activated for 15 s to evaporate the acetone from the acetone–D_2_O mixture present in the NMR tube (see Supporting Information 3). A further 5 s delay is needed to inject 100 μL of buffer solution to adjust the aqueous pH to circumneutral values and obtain isotonicity. In the last step, a volume of 200 μL of H_2_O_2_, the enzymatic, or the cell solution—in different experiments—is injected through a different plastic cannula. The carbon spectrum is acquired *via* the subsequent application of 20° (45° for HeLa cell experiments) flip angle pulses every 2 s.

### MINERVA

**Figure 3:**
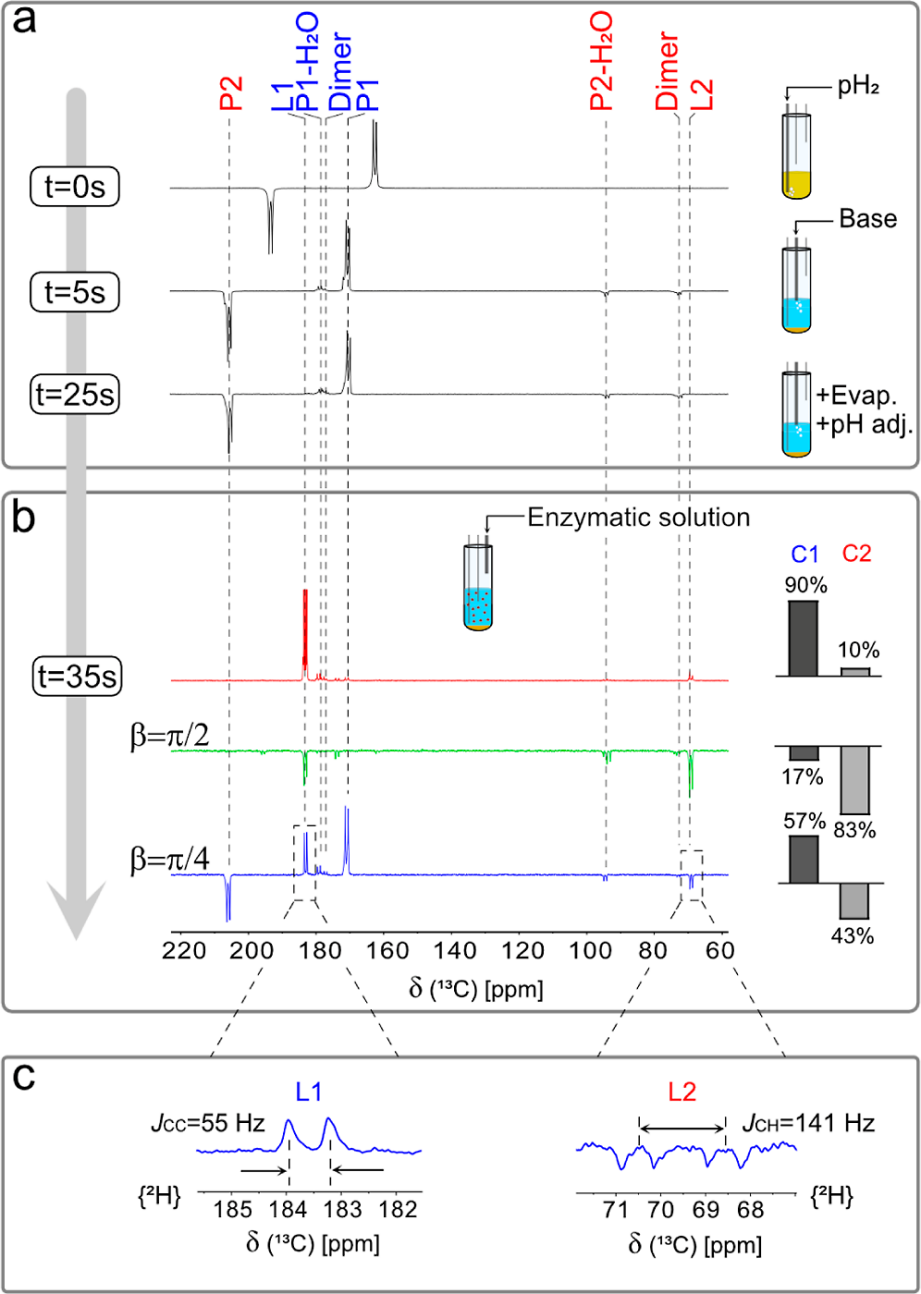
(a) (1) ^13^C NMR of the precursor after hydrogenation (*t* = 0 s), after reaction with 50 mM Na_2_CO_3_ (*t* = 5 s), and after vacuum evaporation and pH adjustment (*t* = 25 s). (b) ^13^C NMR of HP [1,2-^13^C] P–L conversion after injection of 200 μL of 100 LDH units (*t* = 35 s) acquired to enhance C1 (red spectrum → only first block of MINERVA to produce *S*_1y_), to enhance C2 (green spectrum → full MINERVA with β = π/2 to produce *S*_2y_), and to enhance C1 and C2 (blue spectrum → full MINERVA with pi/4 to produce −0.7 × *S*_1y_ and + 0.7 × *S*_2y_). Bar graphs indicate the fraction of the signal with C1 (positive phase) and C2 (negative phase) characteristics. (c) Expanded L_1_ and L_2_ regions with {^2^H} decoupling. The vertical gray lines indicate δP_1_ = 170.5 ppm (162.3 ppm before cleavage) and δP_2_ = 205.6 ppm (193.2 ppm before cleavage). The hydrated forms of pyruvate, P_1_-H_2_O and P_2_-H_2_O, resonate at 179.7 and 95 ppm, respectively. Dimer forms of pyruvate at δ = 177.3 and δ = 73.0 ppm. δL_1_ = 183 ppm and δL_2_ = 69 ppm after injection of LDH.

We describe the hyperpolarized precursor molecule as a four-spin system with two parahydrogen ^1^H, *I*_1_ and *I*_2_, and two ^13^C nuclear spins *S*_1_ and *S*_2_, with the relevant *J*-coupling network reported in Figure 1a. The initial state upon hydrogenation at 7T is incoherently averaged to ρ_1_ = 2 *I*_1z_*I*_2z_. The first block of the sequence (1–2 in Figure 2a) converts ρ_1_ primarily into in-phase magnetization on the C1 carbon to yield ρ_2_ = *S*_1y_ which evolves through the second block (2–3 in Figure 2a) to ρ_3_ = −cos(β)*S*_1y_ + sin(β)*S*_2y_. By varying the β angle, the signal can be filtered at C1 (first block), C2 (β = π/2), or both carbon positions (β = π/4) (see Figures 2b and S8). After MINERVA (β = π/4) and base injection, the pyruvate chemical shifts jump from δP_1_ = 162.3 ppm to δP_1_ = 170.5 ppm for C1 and δP_2_ = 193.2 ppm to δP_2_ = 205.6 ppm for C2 (see Figure 3a). Each peak is further split by *J*_CC_ ∼ 68 Hz. The ^13^C polarization levels (Pol) at each steps go from Pol ∼ 24 ± 1.6% before cleavage to Pol ∼ 7 ± 1.0% after cleavage, Pol ∼ 2.5 ± 1.0% after solvent evaporation and buffer pH adjustment, to the final average Pol< ∼ 1.0 ± 0.3% at the moment of H_2_O_2_ or enzymatic/cell solution injection (Figure 3a). The polarization levels are similar at C1 and C2. The values of *T*_1_ at 7T and 55 °C before and after cleavage are *T*_1_ ∼ 79.5 s and *T*_1_ ∼ 63.9 s at C1 and *T*_1_ ∼ 66.7 s and *T*_1_ ∼ 45.2 s for C2. We note that based on *T*_1_, we would expect a ∼40% drop in polarization rather than ∼70% after cleavage. The total volume, before solvent evaporation, of ∼200 μL is still within the coil region, and no losses in signal are expected. The extreme pH before buffer adjustment can be related to the polarization loss. However, we have not tried here to investigate further this aspect. We only note that the pyruvate instantaneous polarization can also be approximately estimated *via* the asymmetry of the corresponding ^13^C doublets as previously shown.^49,55^

### *In Vitro* Enzymatic Reaction Monitoring

In the final step, 200 μL of a buffer solution containing 100 units of rabbit muscle LDH and 20 mM NADH, thermalized at 37 °C, is injected at 7T, and the P–L conversion was monitored by 20° ^13^C pulses every 2 s (Figure 3b). For lactate, the resonances δL_1_ = 183 ppm and δL_2_ = 69 ppm are observed. In Figure 3b, MINERVA is adapted to retain the signal primarily on C1 (red spectrum using the first block of the sequence, *i.e.*, 1–2 in Figure 2a), on C2 (green spectrum by β = π/2), and on both (blue spectrum by β = π/4). The expansion in Figure 3c shows the {^2^H}^13^C spectrum at the final step, where *J*_CH_ = 141 Hz splitting at 69 ppm points out L_2_, whereas *T*_1_ for P_1_ and P_2_ is similar (∼17 s of apparent decay at 55 °C), and the *T*_1_ ratio for L_1_ and L_2_ is about 3.0 (*i.e.*, ∼7 s of apparent decay at 55 °C for L_2_). The faster L_2_ decay is partially explained by the spatial proximity of the lactate ^1^H leading to a stronger dipolar contact. The enzymatic reaction was also monitored at 37 °C (50 LDH units). The fitting model used, introduced by Khegai and *et al.*,^56^ assumes two exchanging pools with a unidirectional *k*_PL_: . It has been previously shown that back conversion is typically negligible^57^ (see Supporting Information 5). The advantage and limitation of the model used is that the different metabolic relaxation rates are included in a single effective rate *R*_eff_, and *k*_PL_ is obtained by a simple to compute pseudoinversion matrix operation. The table in Supporting Information 6 shows agreement between the *k*_PL_ values measured *via* C1 and C2 in three replicate experiments at different conditions.

### In-Cell Real-Time P–L Conversion

**Figure 4:**
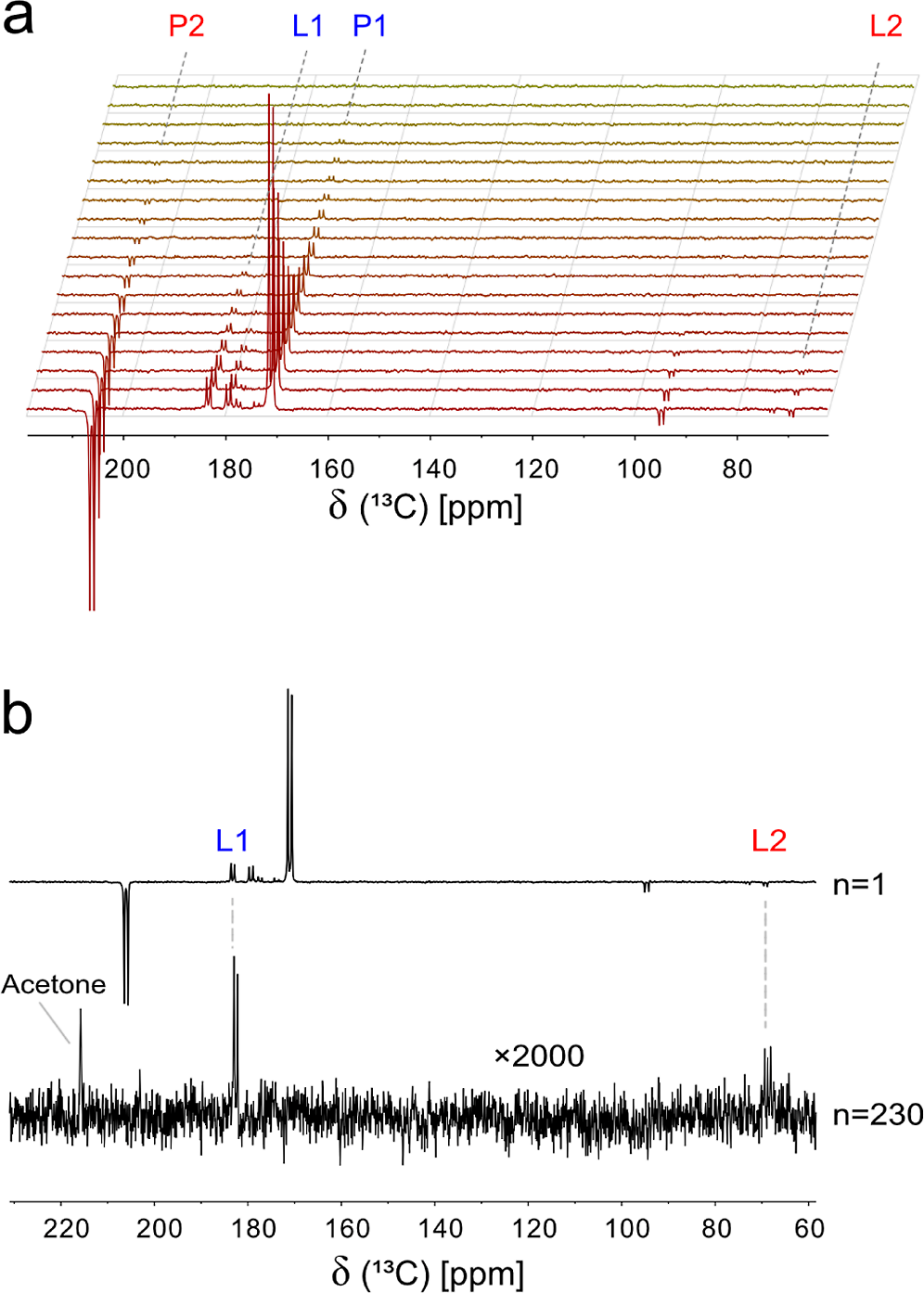
(a) Pseudo-2D ^13^C NMR experiments with P–L conversion detected in HeLa cells (∼25 M) at 7T, with cells kept at 37 °C until injection. (b) ^13^C hyperpolarized NMR spectrum (ns = 1, 45° recording angle) and thermal spectrum (ns = 230, 90°, ×2000-fold) acquired from the Hela cell sample.

Figure 4a shows that the P–L conversion can be followed through both carbon signatures C1 and C2, also *in-cells*. HeLa cancer cells are prepared as described in the Supporting Information. After the polarization steps detailed in Figure 3a, 200 μL of a HeLa cell slurry (∼25 millions) in fresh culture medium (DMEM with supplements as per Supporting Information 3) is injected at 7T, and the ^13^C spectrum is acquired *via* consecutive {^1^H, ^2^H}^13^C 45° pulses starting approximately ∼8–10 s after cell injection. In addition to the pyruvate, hydrated form, and dimer signals, the C1 and C2 signatures of lactate at 183.2 and 69.2 ppm are visible in Figure 4a,b. The experimental chemical shifts are summarized in Table 1.

**Table 1:** ^13^C Chemical Shifts for the In-Cell Real-Time P–L Conversion at pH 7, *T* = 37 °C

	experimental chemical shifts, ppm	
	^**13**^**C1**	^**13**^**C2**
pyruvate	171.0	206.0
hydrate	179.3	94.6
dimer	177.3	73.0
lactate	183.2	69.2

The thermal spectrum in Figure 4b indicates the presence of ∼5 μL residual acetone-*d*_6_ (see Supporting Information 7). The cell viability at the end of the experiment is conservatively about >90%. Although following P–L conversion *via* MINERVA (β = π/4), comes at the cost of splitting the signal between C1 and C2, the combined effect of a doubly labeled pyruvate and a tunable sequence gives the highest degree of flexibility.

### Factors Influencing ^13^C Polarization

At every step in Figure 3, the polarization is affected by many parameters such as H_2_ solubility, the solvent, the catalyst, and others. Acetone has a relative low toxicity^58^ and guarantees a good H_2_ solubility and a weak binding affinity to the Rh(I) catalyst used. This, in turn, favors an improved efficiency and better polarization levels as the fast displacement of the product molecule from the metal center limits the singlet/triplet mixing on the intermediate reaction products that has been linked to loss of polarization.^59,60^ We further note that for the catalyst in acetone-*d*_6_ a turnover frequency (*i.e.*, the moles of substrate that a mole of catalyst can convert per second) higher than in other common organic solvents is observed.^60^ In addition, the acetone boiling point, 58 °C, is compatible with the evaporation step we have implemented. Deuteration of the initial precursor is instrumental to polarization transfer achieved by MINERVA by restricting the effective nuclear spin system and minimizing dilution of signal enhancement. Alternative side arms are possible and were recently investigated by our group.^54^ According to our studies, the precursor vinyl pyruvate granted the highest levels of carbon hyperpolarization in [1-^13^C]pyruvate so far (*i.e.*, 59.7 ± 2.5 and 27 ± 1% on the precursor molecule and free pyruvate, respectively)^54^ and was therefore the precursor of choice in the current investigation. The [1,4-Bis(diphenylphosphino)butane](1,5-cyclooctadiene)rhodium(I) tetrafluoroborate Rh catalyst is frequently used in PHIP experiments for two main reasons: (i) it is commercially available and (ii) it enables the pairwise addition of parahydrogen, which is necessary to preserve the parahydrogen’s initial spin character. In the present work, we did not attempt to compare multiple Rh catalysts. However, we note that recently it has been reported that structural modifications of the Rh catalyst used here lead to improved catalytic activity, faster hydrogenation reaction, and possibly improved ^13^C polarization.^61^

### Real-Time Pyruvate Decarboxylation Monitoring

**Figure 5:**
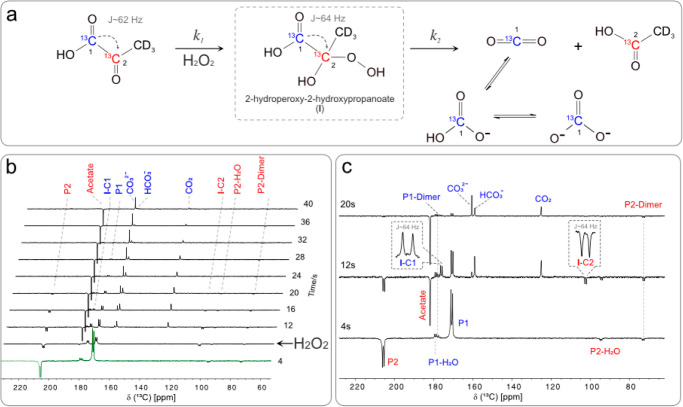
(a) Non-enzymatic decarboxylation reaction between [1,2-^13^C]pyruvate (5 mM) and H_2_O_2_ (326 mM) *via* the intermediate formation of 2-hydroxyperoxy-2-hydroxypropanoate (**I**) at pH 7. The C1 and C2 ^13^C positions are indicated in blue and red, respectively. (b) Series of ^13^C hyperpolarized NMR spectra (20-degree flip angle). The green spectrum at 4s shows hyperpolarized [1,2-^13^C]pyruvate. From 8s, after the injection of H_2_O_2_, the spectra report on the H_2_O_2_-induced [1,2-^13^C]pyruvate decarboxylation reaction. Species deriving from C1 and C2 are indicated vertically in blue and red, respectively. (c) 1D ^13^C NMR extracts from the pseudo 2D experiment in (b) before (bottom) and after (middle and top) the injection of H_2_O_2_. Evidence of **I** at 12 s *via* C1 and C2 positions at 176.1 and 102.3 ppm with *J*_C1C2_ ∼ 64 Hz. Pyruvate has almost completely been consumed by the reaction in the top spectrum with loss of spectral signature for **I**.

As explained in the Introduction, pyruvate protects neurons and other cell types from the toxic hydrogen peroxide (H_2_O_2_).^53,62^ H_2_O_2_ is stable in abiotic environments at ambient temperature and neutral pH, yet rapidly kills any type of cells by producing highly reactive hydroxyl radicals. Although catalases are commonly deployed by cells as powerful H_2_O_2_ scavengers,^63^ pyruvate is also used to quench H_2_O_2_ by reacting quickly and irreversibly to yield acetate and carbon dioxide. According to a proposed mechanism, supported by experimental results with H_2_^18^O_2_, the product formation in the H_2_O_2_-induced pyruvate decarboxylation occurs through the intermediate 2-hydroxyperoxy-2-hydroxypropanoate (**I**)^64−66^ (see Figure 5a). Low-temperature ^13^C NMR and UV spectrophotometry have been used to capture **I**’s presence.^64,65^ However, the real-time monitoring of **I** at high temperature by NMR has been hampered so far by the low sensitivity and limited lifetime. In Figure 5b, we report the stacked plot showing the ^13^C NMR for the non-enzymatic decarboxylation of [1,2-^13^C]pyruvate according to the reaction in Figure 5a. The experiment has been conducted at 55 °C following the steps already described in Figure 3a. In short, we applied MINERVA to hyperpolarize 5 mM [1,2-^13^C]pyruvate as per Figure 1a. After injecting the buffer solution to bring the pH to 7 (pH 2 and 9 in other experiments, see Supporting Information 6.1), we waited 4 s before applying a series of 20-degree flip angle pulses to track the ^13^C NMR signal. The ^13^C NMR spectra at the bottom of Figure 5b,c show the presence of pyruvate, the corresponding hydrated form, and the dimer signals.^65^ In the final step, after the injection of 200 μL of H_2_O_2_ at 326 mM (H_2_O_2_ 1%), the ^13^C NMR spectrum drastically changes. The middle spectrum in Figure 5c shows a negative and a positive signal both separated into two peaks 64 Hz apart (squared dashed box). The spectral positions are compatible with the previously reported values for **I** in methanol/water in ref (65). These signals are relatively short-lived as they vanish 12 s after the H_2_O_2_ injection, as shown in the top transient (20 s) in Figure 5c. We attribute these two signals to the C1 and C2 signatures of [1,2-^13^C]**I** due to their opposite phases, identical 64 Hz splitting, short lifetime, and compatibility with previously reported values.^65^ In addition to the [1,2-^13^C]**I** formation, we observe the presence of all of the species detailed in Figure 5a–c, whose chemical shift is reported in Table 2 for clarity. The carbon polarization is sufficiently high to investigate the formation of acetate and CO_2_ in a single experiment using the intermediate [1,2-^13^C]**I** and the C2 and C1 carbon signatures of [1,2-^13^C]pyruvate, respectively. Conveniently, the positive and negative NMR signals correspond to the carbon spins at C1 and C2, respectively. Therefore, different carbon sites are useful for investigating different simultaneous chemical processes.

**Table 2:** ^13^C Chemical Shifts for the Various Species in the Pyruvate Decarboxylation Reaction at pH 7, *T* = 55 °C

	experimental chemical shifts, ppm	
	^**13**^**C1**	^**13**^**C2**
pyruvate	170.6	205.6
hydrate	178.8	94.4
dimer	177.6	72.6
intermediate (**I**)	176.1	102.3
acetate		181.4
bicarbonate	159.0	
carbonate	160.5	
carbon dioxide	125.0	

By using the pathway in Figure 5a as a guide, a fitting model for the reaction rates *k*_1_ and *k*_2_ was developed. The model (see Supporting Information 6) is oversimplified because (i) we omit the pyruvate equilibria of its hydrate and dimer forms as they have the weakest signal in our spectra; (ii) we treat carbon dioxide, bicarbonate, and carbonate as a single product since the equilibria among them is irrelevant for the intermediate formation; and (iii) we assume unidirectional reaction rates. From the analysis detailed in the Supporting Information, assuming a *T*_1_ = 50 s for pyruvate (the same at C1 and C2, as per conventional NMR under similar conditions), *T*_1_ = 3.0 s for **I** (the same at C1 and C2, as different values seem to be less compatible with our experimental data), we find that *k*_1_ = 0.18 s^–1^ and *k*_2_ = 0.23 s^–1^ with a *T*_1_ = 25 s for acetate at 7T. Furthermore, the signals from CO_2_ and HCO_3_^–^ in Figure 5b have similar lifetimes, and as can be seen in Figure 5c, they show comparable polarization levels at each transient. Under these premises and taking into account the fast exchange regime between CO_2_ and HCO_3_^–^, a stable pH is found throughout the chemical reaction by taking the integral ratio of HCO_3_^–^ and CO_2_ according to the Henderson–Hasselbalch equation (eq. Supporting Information 9) at each transient (Figure S15). Additional experiments were conducted at pH = 2 and pH = 9 (see Supporting Information 6.1). At pH = 2 in particular, we see that HCO_3_^–^ forms from ^13^CO_2_ as the bicarbonate at ∼159 ppm signature is only visible after carbon dioxide formation at ∼123 ppm (carbon signals in the dashed gray box in Figure S14b). At basic conditions (pH = 9), pyruvate is immediately quenched by reacting with H_2_O_2_, and we neither observe the formation of **I** nor of ^13^CO_2_ (Figure S14a).

## Conclusions

In conclusion, we succeeded in hyperpolarizing [1,2-^13^C]pyruvate *via* PHIP-SAH. We investigated the P–L metabolic conversion kinetics in aqueous solution *in vitro* and *in-cells* at 7T. To the best of our knowledge, although previous reports on doubly labeled pyruvate hyperpolarization exist,^48,50,52^ no measure of the kinetics *via* C1, C2, or both was reported so far by PHIP-SAH-based techniques.

We also follow in real-time the non-enzymatic pyruvate decarboxylation through different carbon sites C1 and C2, confirming the intermediate formation of 2-hydroperoxy-2-hydroxypropanoate (**I**) that was previously reported only by low-temperature ^13^C NMR and not in real time.

In the proposed experiment, the pH of the solution can be monitored in real time by tracking the production of H^13^CO_3_^–^ and ^13^CO_2_ during the decarboxylation reaction *via* the correspondent carbon signals.

We think that the possibility to follow various chemical reactions or the same reaction *via* different carbon sites, as we show here, without the need of numerous metabolic probes *via* a straightforward and widely accessible PHIP-SAH protocol is particularly noteworthy. The NMR pulse strategy used is flexible and allows for the selective or simultaneous hyperpolarization of C1 and C2 with opposite phase by only changing a single pulse in the MINERVA sequence. We show that the kinetics information can be extracted for P–L conversion from either C1 or C2 site. This approach may also be of use to better analyze mixtures of metabolites that are tagged with different phases in the same sample.

## Supporting Information Available

Chemical synthesis, enzyme and cell experiments, hyperpolarization experiments, kinetic fitting, and simulations (PDF)

## Abbreviations

MFC: magnetic field cycling
PHIP: parahydrogen-induced polarization
SABRE: signal amplification by reversible exchange
MINERVA: maximizing insensitive nuclei enhancement reached *via* para-hydrogen amplification
P–L: pyruvate to lactate
