# Supplementary material for: Real-Time Pyruvate Chemical Conversion Monitoring Enabled by PHIP
Source: J Am Chem Soc. 2023 Mar 1;145(10):5864–71. doi: 10.1021/jacs.2c13198 (PMC10021011; doi:10.1021/jacs.2c13198)
Supplement: Supplementary file 1 — ja2c13198_si_001.pdf [file ja2c13198_si_001.pdf]

SUPPLEMENTARY INFORMATION

Real-time pyruvate chemical conversion monitoring enabled by PHIP

Gabriele Stevanato[a,b], Yonghong Ding[a,b], Salvatore Mamone[a,b], Anil P. Jagtap[a,b], Sergey Korchak[a,b], Stefan Glöggler*[a,b]

[a]: NMR Signal Enhancement Group, Max Planck Institute for Multidisciplinary Sciences, Am Fassberg 11, 37077 Göttingen, Germany.

[b]: Center for Biostructural Imaging of Neurodegeneration of the University Medical Center Göttingen, Von-Siebold-Str. 3A, 37075 Göttingen, Germany

Contents

1. Synthesis of 1,2-^{13}C pyruvate precursor 2
2. LDH solution and Cell preparations 2
3. Production of rapidly signal-enhanced 1,2-^{13}C pyruvate 3
4. Simulations 4
   4.1. Spin system, parameters and Hamiltonian: 5
   4.2. Pulse Sequence: 6
   4.3. Carbon Spectra 7
   4.4. Transformation amplitudes 9
5. Kinetic analysis 10
6. Pyruvate H_2O_2-induced decarboxylation 13
   6.1. Pyruvate decarboxylation at different pH 15
7. Estimation of Lactate hyperpolarization and acetone level 17
8. References 18

1. Synthesis of 1,2-^{13}C pyruvate precursor

Vinyl compounds provide an excellent basis to produce signal-enhanced metabolites, for instance pyruvate, via PHIP effect but their synthesis via transesterification were mostly obtained in low yields. Our recent publication (Ding et. al. 2022) reported a procedure to synthesize 1,2-^{13}C labeled vinyl pyruvate in high yields (80%) as shown in Figure-SI 1. Here briefly, deuteration of the starting material 2-oxopropionic-1,2-^{13}C-acid (1) was performed in presence of D_2SO_4/D_2O to yield 2, which was then treated with trimethyl orthoformate to obtain dimethyl ketal protected derivative (3) in quantitative yields. Installation of the photo-labile protecting group was performed by coupling of diol (4) with dimethyl ketal protected derivative (3) in toluene to yield 5 with moderate yield. Pd-catalyzed transesterification of 5 with deuterated vinyl acetate (6) was performed to obtain 7 with 81% yield (based on the recovery of 5). Deprotection of 7 containing photo-labile protecting group was carried in presence of LED lamp (365 nm) to obtain desired compound (8) with 12% yield.

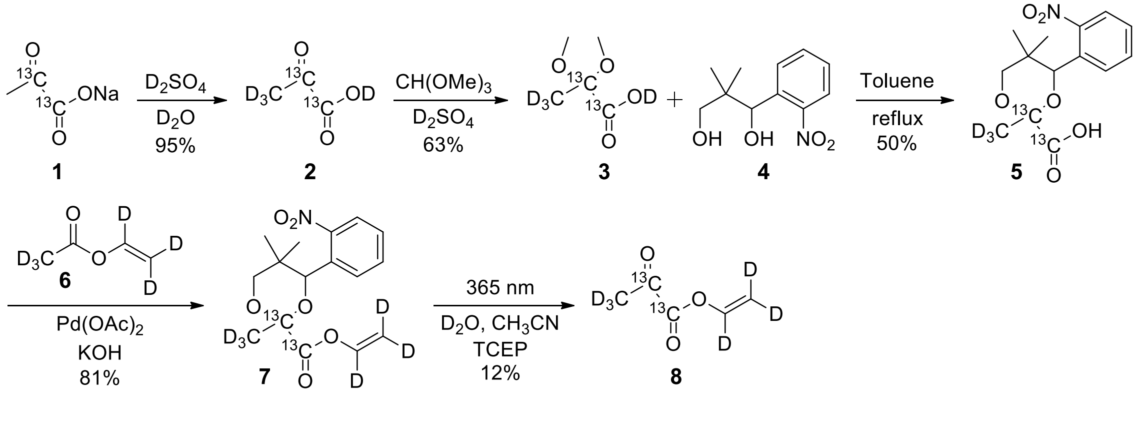

*Figure-SI 1: Synthesis of 1, 2-^{13}C-vinyl pyruvate (**8**)*

2. LDH solution and Cell preparations

To probe the pyruvate-to-lactate conversion *in vitro*, L-lactic dehydrogenase (LDH) from rabbit muscle in ammonium sulfate suspension (sigma, USA) was diluted to 250 or 500 units/ml against protonated buffer of 100 mM HEPES (Sigma, UK), 20 mM NADH (Biomedicals, Japan) and 120 mM NaCl (Roth). Around 200 μL LDH solution (50 or 100 units) was used for one PHIP experiment.

For probing intracellular pyruvate-to-lactate conversion, Hela Kyoto wild-type cells was cultured in the Dulbecco's Modified Eagle Medium supplemented with 4.5 g/L D-Glucose, 2 mM glutamine, 1 mM Sodium Pyruvate, 10% (v/v) heat-inactivated fetal bovine serum, 100 U/mL Penicillin and Streptomycin (all the components were purchased from Thermo-Fisher scientific). The cells were grown at 37°C in a humidified 5% CO_2 atmosphere to around 70-80% confluency before collection. Before the PHIP experiment, Hela cells were detached from the cell culture flasks using trypsin/EDTA (0.05%/0.02%), centrifuged under 130 × g for 3 min at room temperature, washed once with PBS and counted on a

haemocytometer. Around 25 million cells were collected and re-suspended into 120 μL DMEM medium containing 50 mM NADH for one PHIP experiment.

3. Production of rapidly signal-enhanced 1,2-^{13}C pyruvate

A purity of 99% pH_2 was generated using a He-cooled pH_2-generator (generator T=20 K), equipped with a Cryocooler system (Sumimoto HC-4A helium compressor, Sumimoto Cold Head CH-204 with ph_2 reaction chamber by ColdEdge Technologies), temperature controller (Lake Shore Cryotronics, Inc.) and home built valve and tubing system. 5 mM of hydrogenation catalyst ([1,4-bis(diphenylphosphino) butane] (1,5-cyclooctadiene)rhodium(I) tetrafluoroborate) was dissolved in acetone-d6. For each sample, the vinyl precursor was added to 0.1 ml of the catalyst solution to obtain a concentration of 5 mM. Samples were placed in a 5mm NMR-tube, degassed by bubbling N_2 gas for 1 minute and hydrogenated at 7 bar for 20 s by bubbling using a home-built setup (Fig. SI 2) inside a probehead of 7 T Bruker spectrometer (Avance III) heated to 328 K. By using the pulse sequence MINERVA[1] (Figure SI 2), the ^{1}H polarization is transferred to the 1- and/or 2-^{13}C moiety of the pyruvate precursor. Schematics and pictures of the gas delivery and organic solvent evaporation set up are provided at Fig. SI 2:

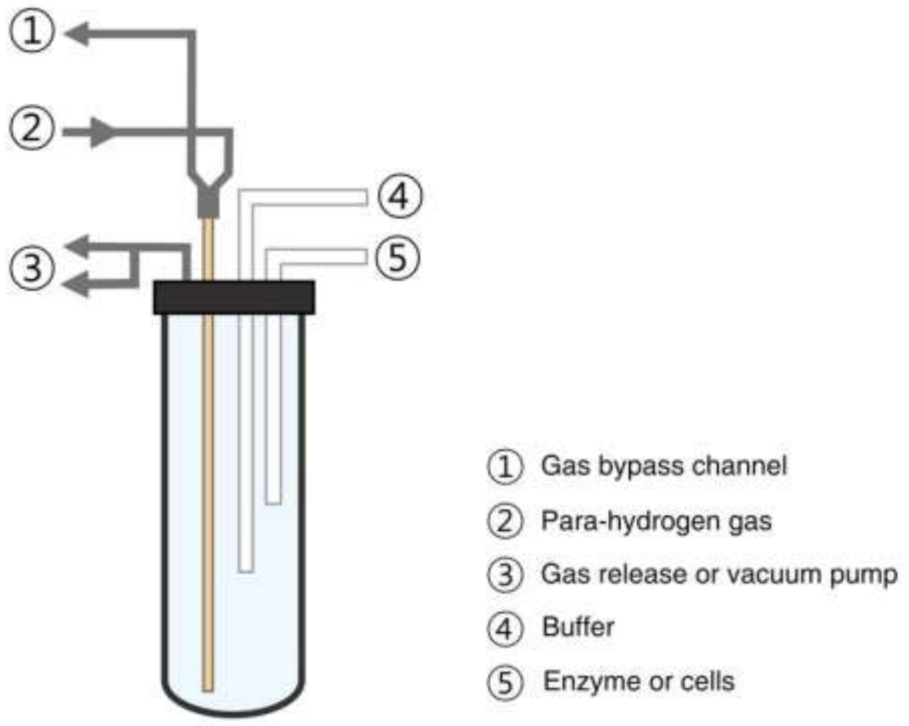

Figure-SI 2: Schematic representation bubbling setup.

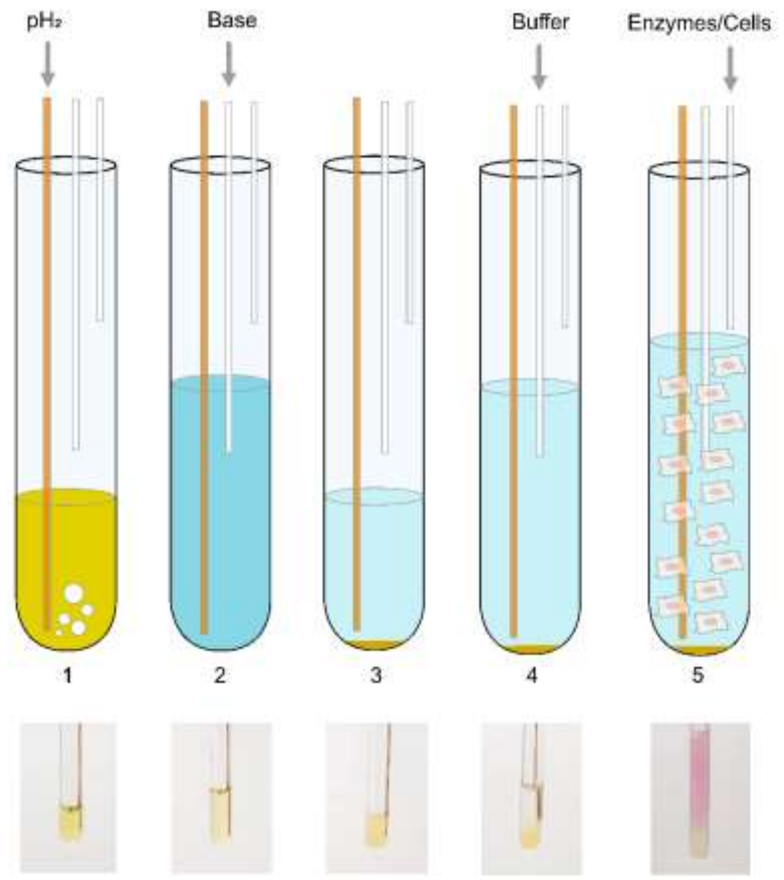

Figure-SI 3: Schematic representation of the protocol used: (1) The acetone solution containing 100 uL of 5 mM [1,4-bis(diphenylphosphino) butane] (1,5-cyclooctadiene)rhodium(I) tetrafluoroborate catalyst and 10 mM [1,2-^{13}C]vinyl pyruvate is bubbled with 98% parahydrogen for 20 seconds through an orange glass fiber (left side). At the end of the hydrogenation period the pulse sequence transfer is applied. (2) Injection via a middle line 0.8 mm i.d. cannula of 100 uL of 50 mM Na_2CO_3 in D_2O (central side). (3) Acetone-d6 removal through a vacuum pump connected with the system. (4) Injection of 100 uL of the buffer solution to neutralize the pH. (5) Injection of 100-200 uL LDH Enzyme or HeLa cells through a different injection line (extreme right). The orange part at the bottom of the NMR tube in (3), (4) and (5) represents the Rhodium catalyst, which tends to precipitate after acetone removal. Pictures of the different stages of the protocol at the bottom.

When polarization transfer was completed, 0.1 ml of 50 mM Na_2CO_3 in D_2O (at room temperature) was added into the NMR tube via a cannula to cleave off the sidearm (Figure-SI 3), followed by 10-12 s of acetone evaporation with a vacuum pump. Then, 0.1 ml of isotonic buffer (0.274 M NaCl, 0.0054 M KCl, 0.0236 mM Na/PO4) was added to adjust the pH of the mixture to around 7.4 followed by addition of around 25 millions Hela cells via a cannula. ^{13}C spectra were recorded with a flip angle of 45° and a repetition delay of 2 s.

4. Simulations

The J coupling parameters have been determined via the 1D carbon 13 spectra and proton NMR spectra. The parameters have been used to simulate the effect of the pulse sequence as shown in the following figures-SI 4-8 using Spindynamica[2] package for Mathematica.

4.1. Spin system, parameters and Hamiltonian:

[1,2-13C]pyruvate

Spin System

```
SetSpinSystem[4]
··· SetSpinSystem: the spin system has been set to {{1, 1/2}, {2, 1/2}, {3, 1/2}, {4, 1/2}}
··· SetBasis: the state basis has been set to ZeemanBasis[{{1, 1/2}, {2, 1/2}, {3, 1/2}, {4, 1/2}}, BasisLabels → Automatic].
```

Precursors Parameters

```
B0 = 7;
δ1 = 4.41 × 10^-6;
δ2 = 4.70 × 10^-6; (* not important for the carbon spectrum*)
δ3 = 160.7 × 10^-6;
δ4 = 191.8 × 10^-6;
J12 = 7.13;
J13 = 3.0;
J23 = 0.3;
J34 = 68.3;
```

Hamiltonian

```
HZ = 2 π (MagnetogyricRatio[1] B0)/(2 π) δ1 opI[1, "z"] + 2 π (MagnetogyricRatio[1] B0)/(2 π) δ2 opI[2, "z"] +
  2 π (MagnetogyricRatio[13] B0)/(2 π) δ3 opI[3, "z"] + 2 π (MagnetogyricRatio[13] B0)/(2 π) δ4 opI[4, "z"];
HJ = 2 π J12 opI[1].opI[2] + 2 π J13 opI[1, "z"].opI[3, "z"] + 2 π J23 opI[2, "z"].opI[3, "z"] + 2 π J34 opI[3].opI[4];
Ht = HZ + HJ;
```

Figure-SI 4: SpinDynamica[2] code for the spin system formed by two ^{1}H (spin 1 and 2) and two ^{13}C (spin 3 and 4) with chemical shifts (δ1, δ2, δ3, δ4) and J-coupling parameters (J12, J13, J23, J34).

4.2. Pulse Sequence:

Pulse sequence with a variable β pulse

```
(Debug) In[]:= ESOtoC3C4[β_] := {
    RotationSuperoperator[{1, 2}, {π/2, "y"}],
    {None, 1/(4*J13)},
    RotationSuperoperator[{1, 2, 3, 4}, {π, "x"}],
    {None, 1/(4*J13)},
    RotationSuperoperator[{1, 2}, {π/2, "-y"}],
    {None, 1/(4*J12)},
    RotationSuperoperator[{1, 2}, {π, "x"}],
    {None, 1/(4*J12)},
    RotationSuperoperator[{1, 2, 3, 4}, {π/2, "y"}],
    {None, 1/(4*J13)},
    RotationSuperoperator[{1, 2, 3}, {π, "x"}],
    {None, 1/(4*J13)},
    {None, 1/(4*J34)},
    RotationSuperoperator[{3, 4}, {π, "x"}],
    {None, 1/(4*J34)},
    RotationSuperoperator[{3, 4}, {β, "y"}],
    {None, 1/(4*J34)},
    RotationSuperoperator[{3, 4}, {π, "x"}],
    {None, 1/(4*J34)}
   };
```

Figure-SI 5: Pulse sequence definition with a variable β angle.

4.3. Carbon Spectra

13C spectra

```
(Debug) In[ ]:= NP = 20 * 1024 - 1;
ppmrangeC = 300 × 10^-6;
ppmrangeH = 10 × 10^-6;
SWC = (MagnetogyricRatio[13] B0)/(2 π) ppmrangeC;
SWH = (MagnetogyricRatio[1] B0)/(2 π) ppmrangeH;

(Debug) In[ ]:= sigC[α_] := Signal1D[{{2 π SWC, NP}}, Preparation -> ESOtoC3C4[α], BackgroundGenerator -> Ht,
   InitialDensityOperator -> opI[1, "z"].opI[2, "z"], Observable -> {3, 4}];

(Debug) In[ ]:= samplsC[α_] := Expand[sigC[α]][[All, 2]];
```

Transfer to carbon 4 at 190 ppm

```
(Debug) In[ ]:=
ListPlot[
 Reverse@Re@Fourier[samplsC[π / 2], FourierParameters → {0, 1}],
 PlotRange -> {{150, 200}, All},
 PlotStyle -> {Blue, Thick},
 FrameLabel → {"δ13C (ppm)", "Int"}, LabelStyle → Directive[FontFamily → "Helvetica", Black, FontSize → 20],
 DataRange -> {0, ppmrangeC * 10^6}, GridLines → {{(δ3) * 10^6, (δ4) * 10^6, 160.24, 161.15}, {}},
 GridLinesStyle → Directive[Thick, Dashed, Orange], ImageSize → Large, ScalingFunctions → {"Reverse"}]
```

··· Signal1D: Using SignalCalculationMethod → Diagonalization

··· Signal1D: the last sampling point has been dropped in order to get an even number of points.

··· Signal1D: Using LineBroadening → 2π × 1.60973 rad s^{-1}.

(Debug) Out[ ]=

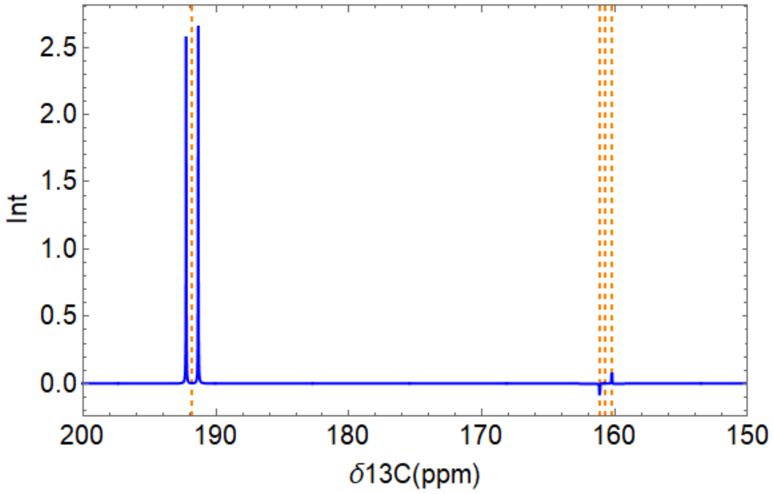

Figure-SI 6: Transfer to C2 (spin number 4 in the pulse sequence definition)

Transfer to both carbons 3 and 4

(Debug) In[▫]:=

```
ListPlot[
 Reverse@Re@Fourier[samplsC[π / 4], FourierParameters → {0, 1}],
 PlotRange -> {{150, 200}, All},
 PlotStyle -> {Black, Thick},
 FrameLabel → {"δ13C(ppm)", "Int"}, LabelStyle → Directive[FontFamily → "Helvetica", Black, FontSize → 20],
 DataRange -> {0, ppmrangeC * 10^6}, GridLines → {{(δ3) * 10^6, (δ4 ) * 10^6, 160.24, 161.15}, {}},
 GridLinesStyle → Directive[Thick, Dashed, Orange], ImageSize → Large, ScalingFunctions → {"Reverse"}]
```

Signal1D: Using SignalCalculationMethod → Diagonalization

Signal1D: the last sampling point has been dropped in order to get an even number of points.

Signal1D: Using LineBroadening → 2π × 1.60973 rad s^-1.

(Debug) Out[▫]=

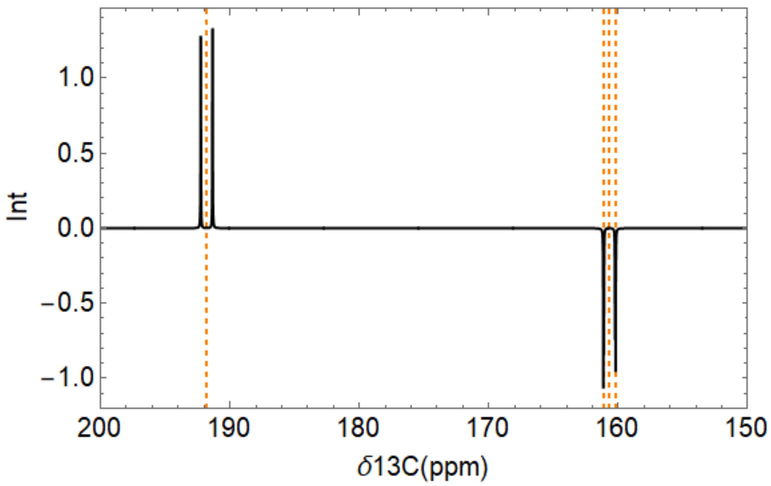

Figure-SI 7: Transfer to C1 and C2 (spin numbers 3 and 4 in the pulse sequence definition) via a sequence with β=π/4.

4.4. Transformation amplitudes

The following code shows the transformation from the initial state upon hydrogenation to the final target state.

Trasformation Amplitudes

Conversion to C1 (spin number 3 in red) or C2 (spin number 4 in black)

```
C3 = Table[
    TransformationAmplitude[
     2 opI[1, "z"].opI[2, "z"] → -opI[3, "y"],
     ESOtoC3C4[α],
     BackgroundGenerator → Ht],
    {α, π / 10000.0, π, π / 20.0}
   ];
```

```
C4 = Table[
    TransformationAmplitude[
     2 opI[1, "z"].opI[2, "z"] → opI[4, "y"],
     ESOtoC3C4[α],
     BackgroundGenerator → Ht],
    {α, π / 10000.0, π, π / 20.0}
   ];
```

Plot

```
ListPlot[
  {C3, C4},
  Joined → True,
  FrameTicks → {{Automatic, None}, {{{1, "0"}, {5.5, "π/4"}, {11, "π/2"}, {16.5, "3π/4"}, {20, "π"}}, None}},
  FrameLabel → {"β flip angle", "Transformation Amplitude"},
  LabelStyle → Directive[FontFamily → "Helvetica", Black, FontSize → 20], PlotStyle → {Red, Black, Thick},
  GridLines → {{}, {}}, ImageSize → Large]
```

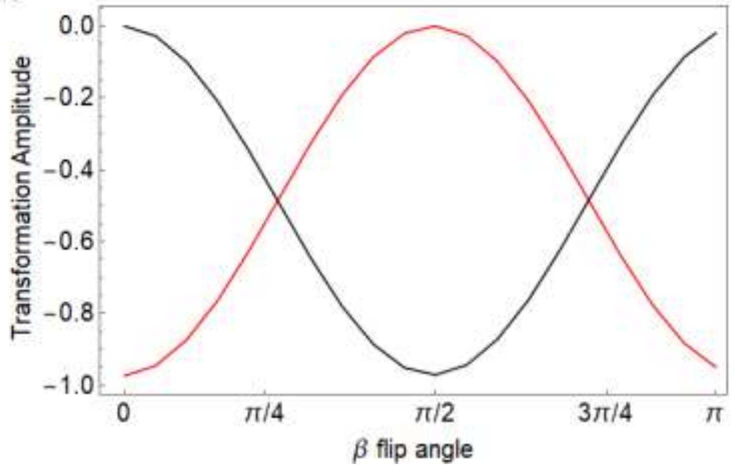

Figure-SI 8: Transformation from the initial operator $2I_{1z}I_{2z}$ into C_{3y} (red) or C_{4y} (black) by varying the flip angle pulse β in MINERVA.

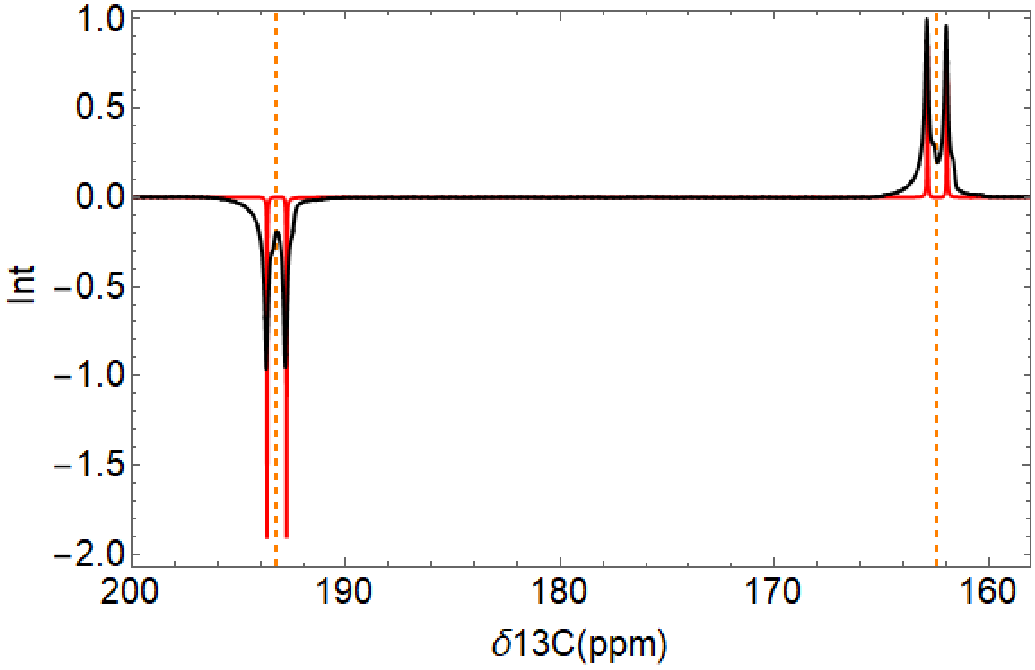

Figure-SI 9: In black is ^{13}C NMR spectrum after parahydrogenation, and pulse sequence application. In red is the simulation spectrum assuming a homonuclear ^{13}C coupling J=68.3 Hz and δ1=162.3 ppm and δ2=193.2 ppm. The orange dashed vertical lines are centered at δ 1 and δ 2.

5. Kinetic analysis

For *in-vitro* enzymatic experiments, the pyruvate-to-lactate reaction is predominantly shifted towards lactate and in accordance with previously reported studies, we assume an unidirectional conversion rate:

$$Pyr \underset{kPL}{\longrightarrow} Lac \tag{SI.1}$$

A set of two differential equations models the variation of the pyruvate and lactate signals over time:

$$\frac{dPyr}{dt} = -k_{PL} \times Pyr - \frac{Pyr}{T_1} \tag{SI.2}$$

$$\frac{dLac}{dt} = k_{PL} \times Pyr - \frac{Lac}{T_1} \tag{SI.3}$$

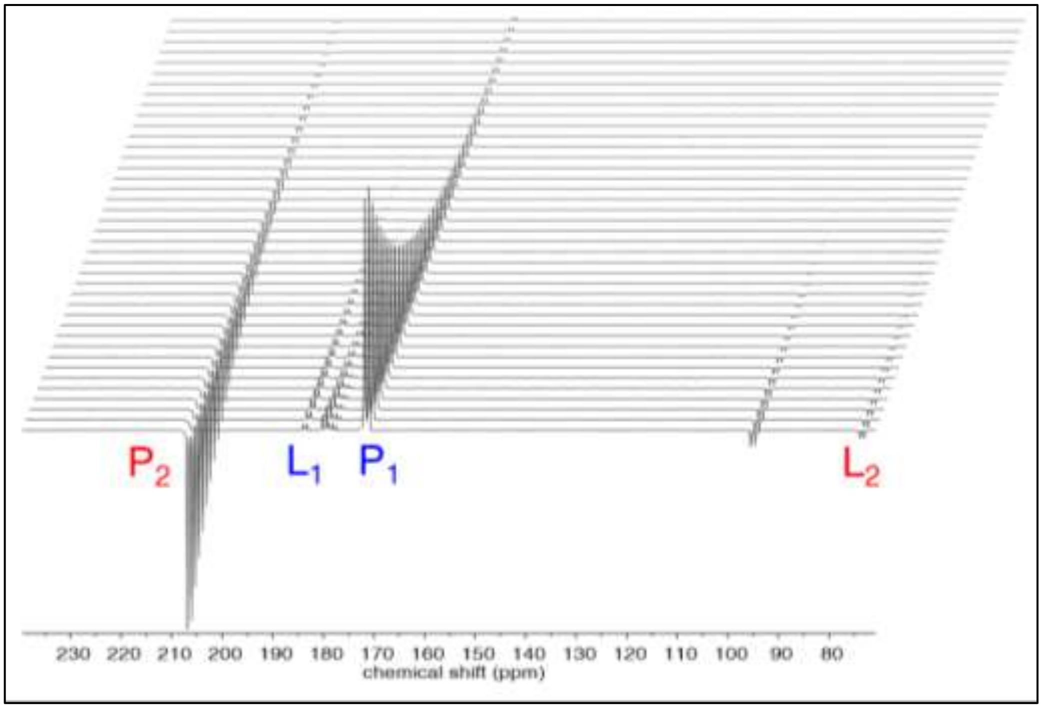

Figure-SI 10: Pyruvate-to-Lactate conversion experiment with 100 units LDH and 20 mM NADH dissolved in 120 mM NaCl and 100 mM HEPES buffer. Every slice is a ^{13}C spectrum probed by a 20° degree flip angle pulse every 2 seconds with proton and deuterium decoupling {^{1}H, ^{2}H}. In blue are the site C1 pyruvate P1 (~170 ppm) and lactate (~183 ppm) L1 carbon signatures. In red show the site C2of pyruvate P2 (~205 ppm) and lactate L2 (~70 ppm) carbon signatures. $T_{1,Lac1}>T_{1,Lac2}$.

Following the discretized version in reference [3] equations SI.2 and SI.3 become:

$$\frac{\Delta L_k}{TR} = k_{PL} \times Pyr_k - R_{eff} \times Lac_k \tag{SI.4}$$

$$R_{eff} = \frac{1}{T_{1,Lac}} + \frac{1-\cos\beta}{TR} \tag{SI.5}$$

The index *k* represent the *k*th slice in the pseudo 2D experiment consisting in the repeated application of β 13C flip angle pulses repeated every *TR*. The effective decay rate *Reff* accounts for signal decay due to Boltzmann thermalization, repetitive excitation with β flip angle pulse (20 degree for *in-vitro* experiments). The pyruvate and lactate integral signal then results in a system of linear equations for k_{PL} and R_{eff} that can be solved by a pseudo matrix inversion:

$$\begin{pmatrix} \frac{\Delta L_1}{TR} \\ \vdots \\ \frac{\Delta L_k}{TR} \end{pmatrix} = \begin{pmatrix} Pyr_1 & -Lac_1 \\ \vdots & \vdots \\ Pyr_k & -Lac_k \end{pmatrix} \cdot \begin{pmatrix} k_{PL} \\ R_{eff} \end{pmatrix} \tag{SI.6}$$

For every slice *k* by the integral values Pyr_k and Lac_k. By pseudoinverting the matrix above k_{PL} and R_{eff} can be estimated. According to SI.5 and SI.6 k_{PL} measured via P1 -> L1 or P2 -> L2 should be identical. However, the effective R_{eff} is expected to be different. The model has been tested on three experiments with 100 LDH units at 55 °C and three experiments with 50 LDH units at 37 °C.

When fitting the data, it must be considered that the number of slices for which L1 and L2 have non-zero values is different, because the relaxation time is different. The presence of noisy points (~0 integral values) leads to a larger imprecision in the fitting routine. In the table below, we used 50 points for the C1 calculations and 10 points for the C2 calculations. The average values at 55 °C are: k_{PL}=0.015 ± 0.001 s^{-1} and k_{PL}=0.013 ± 0.002 s^{-1} for C1 and C2 respectively. At 37 °C k_{PL}=0.030 ± 0.01 s^{-1} and k_{PL}=0.020 ± 0.01 s^{-1} for C1 and C2 respectively. We note that R_{eff} is usually a factor 2 larger for the pyruvate-to-lactate conversion measured at site C2, reflecting the shorter relaxation time. The higher K_{pL} at 37°C than 55°C indicates that 37 °C is a better temperature for LDH enzymatic activity.

55 °C	C1	C2	37 °C	C1	C2
1	k_{PL}= 0.017<br>R_{eff}=0.11	k_{PL}=0.015<br>R_{eff}=0.32	1	k_{PL}= 0.028<br>R_{eff}=0.08	k_{PL}= 0.019<br>R_{eff}=0.19
2	k_{PL}= 0.014<br>R_{eff}=0.15	k_{PL}=0.014<br>R_{eff}=0.33	2	k_{PL}= 0.019<br>R_{eff}=0.06	k_{PL}= 0.023<br>R_{eff}=0.20
3	k_{PL}= 0.014<br>R_{eff}=0.16	k_{PL}= 0.010<br>R_{eff}=0.30	3	k_{PL}= 0.045<br>R_{eff}=0.08	k_{PL}= 0.020<br>R_{eff}=0.15

Table SI-1: Summary of the kPL rates determined for the enzymatic conversions at 55 °C and 37 °C.

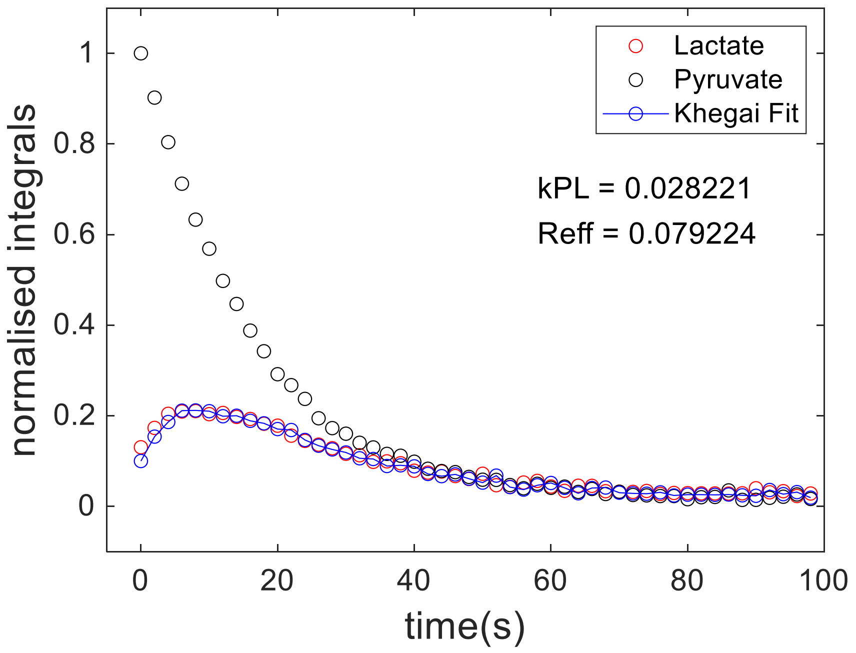

Figure-SI 11: Example of the experimental P-L fitting for P1-L1 at 37 °C. The pyruvate and Lactate integrals are in black and red respectively. The blue points represents the values from the fitting model used.

6. Pyruvate H_2O_2-induced decarboxylation

We model the decarboxylation reaction according to the equations below:

$$P_i \xrightarrow{k_1} I_i \xrightarrow{k_2} Q_i \quad , \quad i = 1,2 \tag{SI.7}$$

$$\frac{dP_i}{dt} = -k_1 P_i - \frac{P_i}{T_{1,Pi}}$$

$$\frac{dI_i}{dt} = k_1 P_i - k_2 I_i - \frac{I_i}{T_{1,Ii}} \quad , \quad i = 1,2 \tag{SI.8}$$

$$\frac{dQ_i}{dt} = +k_2 I_i - \frac{Q_i}{T_{1,Qi}}$$

With initial conditions $P_i(t_0)=Z*P_0$, $I_i(t_0)=I_0$ and $Q_i(t_0)=Q_0$. P_i and I_i indicate the pyruvate and the intermediate integral area at C1 (i=1) or C2(i=2). For Q1 the signals from bicarbonate, carbonate and carbon dioxide have been added. Q2 is the integral area of the acetate peak. The model is oversimplified for the reasons reported in the main text. Nonetheless, it enables a coarse determination of the kinetics constants k_1 and k_2 provided the T_1 values for the different species (P_i, I_i, and Q_i) are assumed and inserted. The equations above have been solved using the Mathematica routine DSolve. A factor $e^{Log(Cos(\beta))/TR}$ with β and TR the flip angle and repetition time used is prepended to the solutions in order to account for the experimental conditions used. We have used the Manipulate routine to match the fitting with the experimental data. The code is provided below.

Differential equations

```
sol = DSolve[{
    P1'[t] == -k1*P1[t] - 1/T1P1*P1[t],
    I1'[t] == k1*P1[t] - k2*I1[t] - 1/T1I1*I1[t],
    Q1'[t] == k2*I1[t] - 1/T1Q1*Q1[t],
    P1[0] == P0, I1[0] == I0, Q1[0] == Q10}, {P1, I1, Q1}, t];
```

Pyruvate

```
pyruvate1[angle_, TR_, P0_, I0_, Q10_, T1P1_, T1I1_, T1Q1_, k1_, k2_, t_] :=
 Exp[Log[Cos[angle Degree // N]]/TR] * e^(-t (1+k1 T1P1)/T1P1) P0
```

Intermediate

```
Intermediate1[angle_, TR_, P0_, I0_, Q10_, T1P1_, T1I1_, T1Q1_, k1_, k2_, t_] :=
 Exp[Log[Cos[angle Degree // N]]/TR] * (e^(-t (1+k2 T1I1)/T1I1 - t (1+k1 T1P1)/T1P1)
   (-e^(t (1+k1 T1P1)/T1P1) I0 T1I1 + e^(t (1+k1 T1P1)/T1P1) I0 T1P1 - e^(t (1+k1 T1P1)/T1P1) I0 k1 T1I1 T1P1 + e^(t (1+k1 T1P1)/T1P1) I0 k2 T1I1 T1P1 +
    e^(t (1+k2 T1I1)/T1I1) k1 P0 T1I1 T1P1 - e^(t (1+k1 T1P1)/T1P1) k1 P0 T1I1 T1P1)) / (-T1I1 + T1P1 - k1 T1I1 T1P1 + k2 T1I1 T1P1)
```

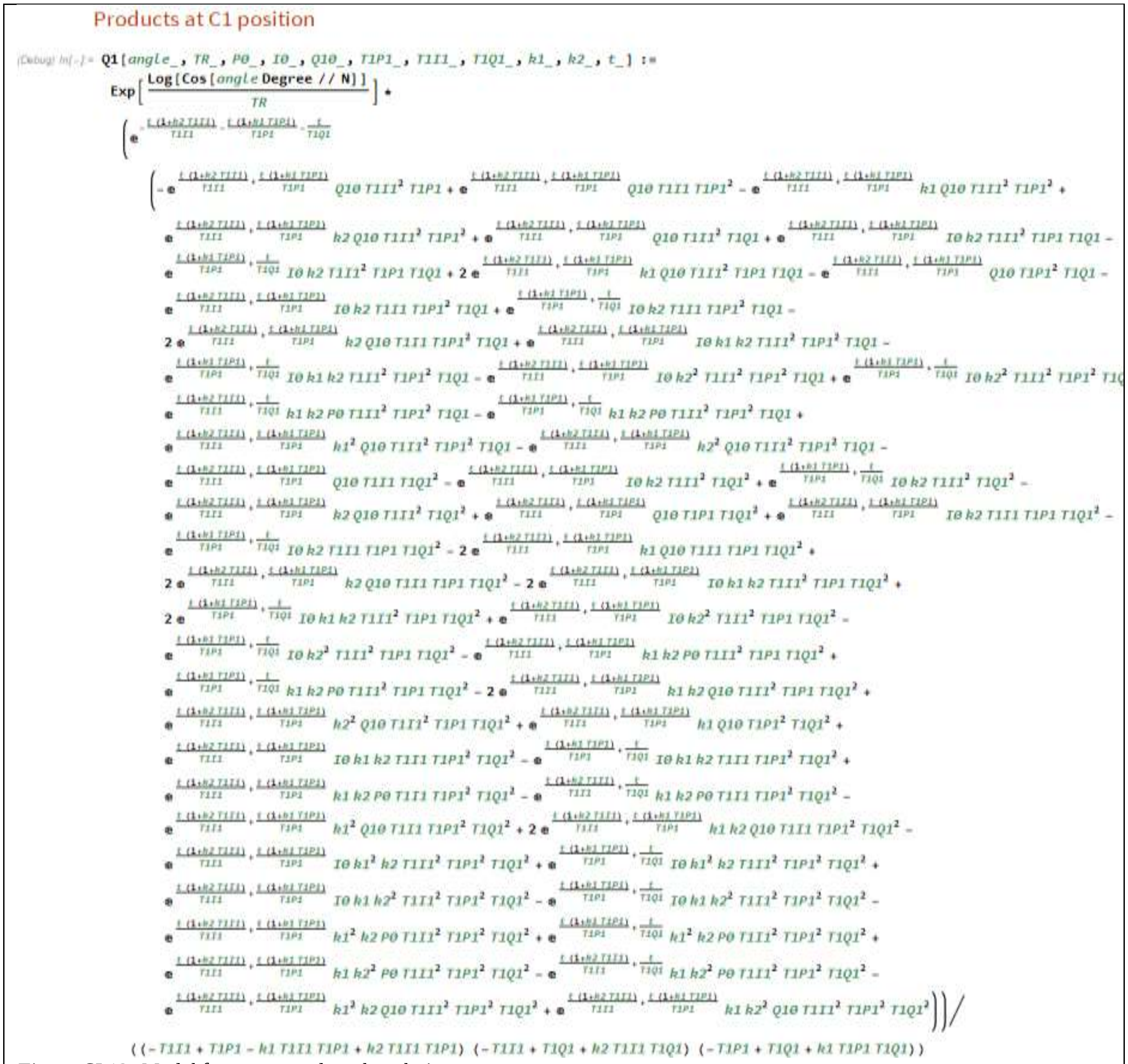

Figure-SI 12: Model for pyruvate decarboxylation

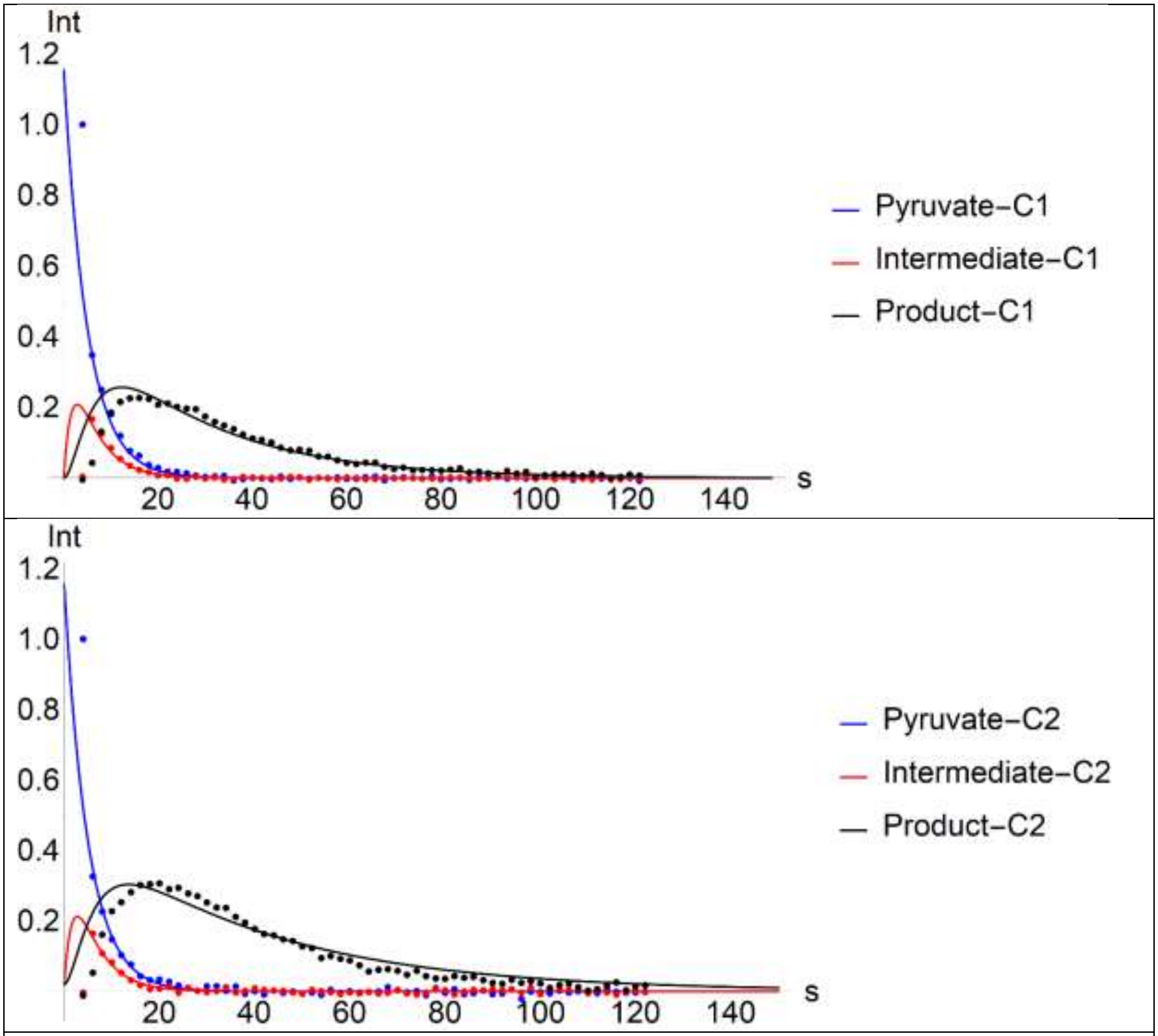

Figure-SI 13: The data points are the integral of the corresponding species at C1 and C2 positions. Product-C1 is the C1 is the integral sum of the signals from HCO_3^-, CO_3^{2-} and CO_2. The product-C2 is the integral value for the acetate resonance. The C1 data points have been fitted with k1 and k2 as free parameters assuming a T1 for Product-C1 of 25s. The C2 data points have been fitted with k1=0.18 s^{-1} and k2=0.232 s^{-1}

In Figure-SI 13 we report the experimental data points and the corresponding fitting curves for pyruvate, the intermediate and the product at C1 and C2 position according to the chemical reaction in Fig. 5a. The product-C1 is the integral sum of the signals from HCO_3^-, CO_3^{2-} and CO_2. The product-C2 is the integral value for the acetate resonance. Following the kinetic model in Fig. SI-12, assuming a T_1=50 s for pyruvate, T_1=3.0 s for **I** and initial conditions I(0)=Q1(0)=0, the fitted values results k_1 and k_2 values are:

Fittings	k_1/s^{-1}	k_2/s^{-1}	T_1,P/s
C1	0.18	0.232	38.0
C2	0.18	0.232	25.0

6.1. Pyruvate decarboxylation at different pH

The same experiment as in Fig. 5 has been conducted at basic (pH=9) and acid (pH=2) conditions. At pH=9 we neither see the intermediate **I** formation, nor the $^{13}CO_2$ signal. Under acid conditions the formation of

HCO_3^- (it only appears from the spectrum some transients later) follows the $^{13}CO_2$ formation (grey box in Figure-SI 14).

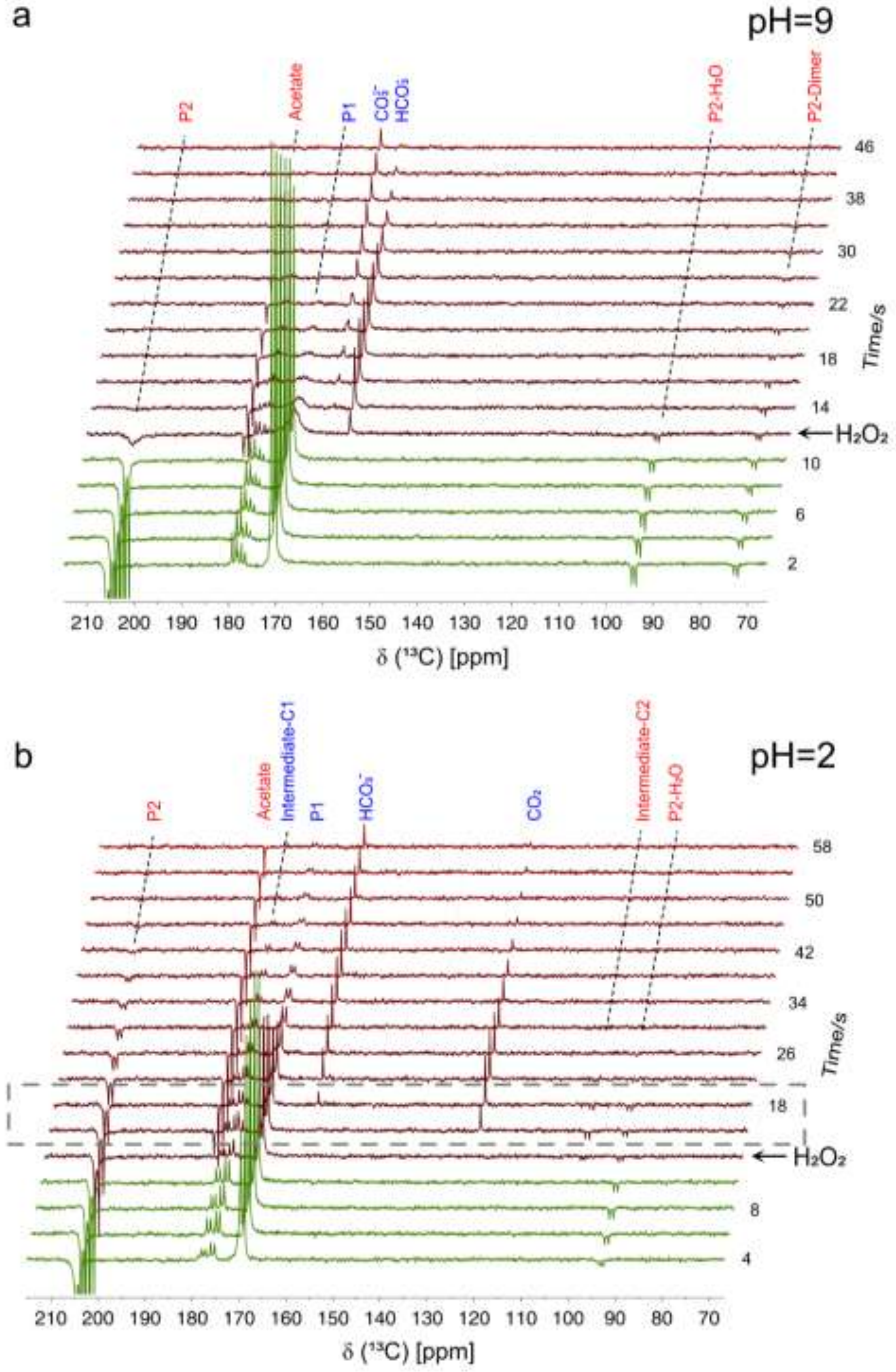

Figure-SI 14: H2O2-induced pyruvate decarboxylation reaction monitored at pH=9 and pH=2

We note that the ability to detect via NMR HCO_3^- and $^{13}CO_2$ signals enables the direct measurement of the bulk pH in solution via the Henderson-Hasselbalch equation:

$$pH = pK_a + log_{10}([HCO_3^-]/[CO_2]) \qquad \text{(SI.9)}$$

We've applied equation (SI.9) to calculate the pH variation during the course of the decarboxylation reaction at around pH 7 by applying the log_{10} function to the ratio of the integrated signals from HCO_3^- and $^{13}CO_2$ at every slice. The HCO_3^- and CO_2 signal intensities in Fig. 6 have been normalized. Their similar trend (* and × curves in blue in Figure-SI 15) indicates a stable pH during the course of the decarboxylation experiments. The red dots are the values returned by equation (SI.9) at each time step in the reaction assuming a pKa=7.1.

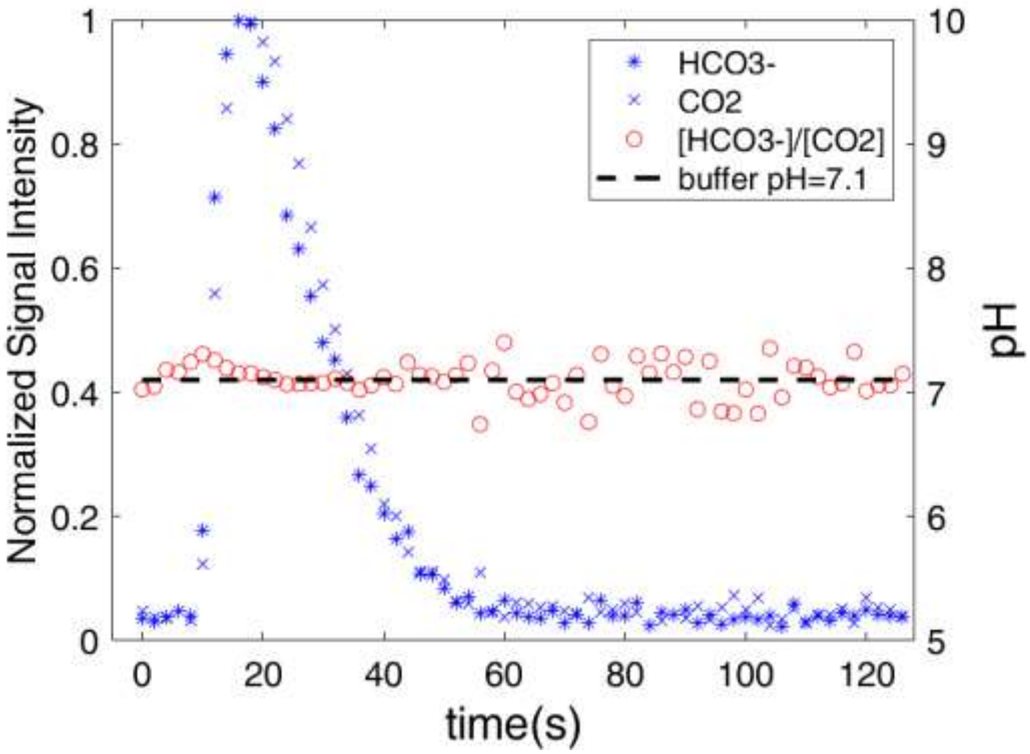

Figure-SI 15: HCO_3^- (158.5 ppm <δ(^{13}C)<159.5 ppm) and CO_2 (124.5 ppm <δ(^{13}C)<125.5 ppm) integral areas in bue (and × respectively) normalized with respect to the correspondent largest integral. The red dots are the pH values during the decarboxylation experiments as per application of equation (1) assuming pKa=7.1. The black dashed horizontal line represents the buffer pH used.*

7. Estimation of Lactate hyperpolarization and acetone level

In Figure 4b we report the thermal and hyperpolarized carbon spectra for the experiment with HeLa cells. By taking the integral values of the acetone and lactate peaks in the thermal experiments we can estimate the final substrate and acetone-d_6 content.

The enhancement and hyperpolarization level is estimated by the following equations:

$$\text{Enhancement factor} = \frac{I\,(hyp.signal)}{I(therm.signal)} \times \frac{rg(therm.)}{rg(hyp.)} \times \frac{ns}{sin\,(flip\ angle)} \qquad \text{(SI.10)}$$

$$\mathrm{P(thermal)} = \tanh\left(\frac{h \cdot \gamma \cdot B_0}{2\pi \cdot 2 \cdot K \cdot T}\right) \quad \text{(SI.11)}$$

$$\mathrm{Hyperpolarization} = \ \mathrm{Enhancement\ factor} \cdot \mathrm{P(thermal)} \quad \text{(SI.12)}$$

In the equations SI 10-12, *I* refers to signal area, *rg* receiver gain, *ns* number of scans, *h* plank constant, *K* Boltzmann constant, *T* temperature, B_0 magnetic field, γ gyromagnetic ratio, flip angle refers to the applied angle before FID detection.

To estimate the level of remaining acetone after cell experiment, the ^{13}C thermal spectrum of the cell sample was recorded. As an external reference, 350 uL of [2-^{13}C] sodium acetate in D_2O at 298K yields an integral I_{ref}=157000 $mol^{-1}*scan^{-1}*rg^{-1}$. By integrating the region at 215 ppm we estimate the final acetone concentration and volume.

$$[Acetoned6] = \frac{13 \times 10^6}{nscan \times rg \times I_{ref}} \times 100 \approx 180 \cdot 10^{-3} \frac{mol}{L}$$

With *nscan*=230 (number of scans); *rg*=203 (receiver gain). The factor 100 accounts for the ~1% natural abundance of ^{13}C. In this experiment, considering that V_{fin}=400 μL, we estimate the volume of acetone in the final solution as:

$$[Acetoned6] \frac{mol}{L} \times 400 \cdot 10^{-6} L \times 64.11 \frac{g}{mol} \times \frac{1}{872} \frac{L}{g} \approx 5 \mu L$$

5uL is the remaining acetone fraction in the final volume 400 μL. This shows that the protocol achieves a consistent, although not complete, reduction of the initial 100 μL acetone volume.

8. References

1. Ding, Y.; Korchak, S.; Mamone, S.; Jagtap, A. P.; Stevanato, G.; Sternkopf, S.; Moll, D.; Schroeder, H.; Becker, S.; Fischer, A.; Gerhardt, E.; Outeiro, T. F.; Opazo, F.; Griesinger, C.; Glöggler, S., Rapidly Signal-enhanced Metabolites for Atomic Scale Monitoring of Living Cells with Magnetic Resonance. *Chemistry–Methods n/a* (n/a), e202200023.

2. Bengs, C.; Levitt, M. H., SpinDynamica: Symbolic and numerical magnetic resonance in a Mathematica environment. *Magn Reson Chem* **2018,** *56* (6), 374-414.

3. Khegai, O.; Schulte, R. F.; Janich, M. A.; Menzel, M. I.; Farrell, E.; Otto, A. M.; Ardenkjaer-Larsen, J. H.; Glaser, S. J.; Haase, A.; Schwaiger, M.; Wiesinger, F., Apparent rate constant mapping using hyperpolarized [1-(13)C]pyruvate. *NMR Biomed* **2014,** *27* (10), 1256-65.
